# Feasibility of parent-mediated behavioural intervention for behavioural problems in children with Autism Spectrum Disorder in Nigeria: a pilot study

**DOI:** 10.1186/s13034-016-0117-4

**Published:** 2016-09-02

**Authors:** Mashudat Bello-Mojeed, Cornelius Ani, Ike Lagunju, Olayinka Omigbodun

**Affiliations:** 1 Child and Adolescent Mental Health Service Unit, Federal Neuro-Psychiatric Hospital, Lagos, Nigeria; 2 Centre for Mental Health, Hammersmith Hospital Campus, Imperial College, London, UK; 3 Department of Pediatrics, University College Hospital, Ibadan, Nigeria; 4 Department of Psychiatry, University College Hospital, Ibadan, Nigeria; 5 Centre for Child and Adolescent Mental Health, University of Ibadan, Ibadan, Nigeria

**Keywords:** Autism Spectrum Disorder, Challenging behaviour, Functional behaviour analysis, Behavioural intervention, Parent education

## Abstract

**Background:**

Autism Spectrum Disorders (ASD) is a disabling and lifelong neuro-developmental disorder. Challenging behaviours such as aggression and self injury are common maladaptive behaviours in ASD which adversely affect the mental health of both the affected children and their caregivers. Although there is evidence-base for parent-delivered behavioural intervention for children with ASD and challenging behaviours, there is no published research on the feasibility of such an intervention in sub-Saharan Africa. This study assessed the feasibility of parent-mediated behavioural intervention for challenging behaviour in children with ASD in Nigeria.

**Methods:**

This was a pre-post intervention pilot study involving 20 mothers of children with DSM-5 diagnosis of ASD recruited from a Child and Adolescent Mental Health Service out-patient Unit. All the mothers completed five sessions of weekly manualised group-based intervention from March to April, 2015. The intervention included Functional Behavioural Analysis for each child followed by an individualised behaviour management plan. The primary outcome measure was the Aggression and Self Injury Questionnaire, which assessed both Aggression towards a Person and Property (APP) and Self Injurious Behaviour (SIB). The mothers’ knowledge of the intervention content was the secondary outcome. All outcome measures were completed at baseline and after the intervention. The mothers’ level of satisfaction with the programme was also assessed. Treatment effect was evaluated with Wilcoxon Signed Rank Tests of baseline and post-intervention scores on outcome measures.

**Results:**

The children were aged 3–17 years (mean = 10.7 years, SD 4.6 years), while their mothers’ ages ranged from 32 to 52 years (mean 42.8 years, SD 6.4 years). The post intervention scores in all four domains of the APP and SIB were significantly reduced compared with pre-intervention scores. The mothers’ knowledge of the intervention content significantly increased post-intervention. The intervention was well received with the vast majority (75 %) of participants being very satisfied and all (100 %) were willing to recommend the programme to a friend whose child has similar difficulties.

**Conclusions:**

Parent-mediated behavioural intervention is a feasible and promising treatment for challenging behaviour in children with ASD in Nigeria. Behavioural intervention should be an integral component in scaling up services for children with ASD in Nigeria.

## Background

Parents of children with Autism Spectrum disorder (ASD) face many challenges in caring for their affected children. The burden is often disproportionately shouldered by mothers [1–3]. Autism Spectrum Disorder (ASD) is a complex and heterogenous disorders with qualitative impairments in social and communication skills, rigid and obsessive interests, and a range of sensory difficulties [4]. In addition to the core social and communication deficits in ASD, challenging behaviour such as aggressive, self-injurious and disruptive problems are common. The prevalence of challenging behaviour varies but reported to be as high as 94 % with aggressive or self-injurious behaviour present in about 55 % of affected children [3, 5, 6]. The presence of challenging behaviour in ASD adversely affects the child, family and the wider society [3, 7]. Challenging behaviour can be a source of major threat to the safety of the affected child and others. It can limit the child’s life opportunities, increase his/her risk of institutionalization and become an obstacle to treatment of core symptoms of ASD. Affected children are socially rejected, stigmatised, at risk of abuse and retaliation from peers, staff and family members [7]. In the absence of appropriate treatment, challenging behaviour in ASD could persist into adulthood with associated developmental and lifelong consequences.

Challenging behaviour also increases the psychosocial stress of care giving especially on mothers who bear a disproportionate burden of care giving [1–3]. The role of mothers as primary care givers places them in a unique position in the delivery of intervention for children with ASD [8, 9]. Involvement of mothers in intervention for their children with ASD has a potential benefit of improved child outcome, reducing associated maternal/family stress, improving care giving skill including identification of possible functions of the aggression [10–13].

Studies suggest that challenging behaviour in ASD could serve a range of functions including a need for attention, protest against unwanted events and access to tangible items [14–16]. Although pharmacological and non-pharmacological approaches are effective for managing challenging behaviour in ASD, behavioural interventions are considered first line [17]. Behavioural interventions are relatively safe and cost effective compared with pharmacological treatments such as anti-psychotics which can have intolerable debilitating side effects [18]. A growing number of studies have demonstrated the benefit of behavioural intervention for challenging behaviour such as aggression in ASD [10, 19, 20]. Notably, studies have shown that as behaviour is influenced by contingencies in the environment, it is similarly sensitive to alteration in such environmental contingencies [14]. Effective behavioural intervention offers important opportunity for improvement for both child and the family caregiver [10, 19, 20].

Despite the good evidence-base for behavioural intervention in managing challenging behaviour in ASD, the main treatment option in Nigeria and other sub-Saharan African countries remains pharmacological [9, 21]. There is virtually no published data on the feasibility of FBA for children with ASD in sub-Saharan Africa. Given the high prevalence of challenging behaviour in ASD and its adverse effect on the affected child, caregiver and the wide society, it is important that appropriate interventions are put in place to identify and address behavioural problems in affected children in sub-Saharan Africa [22, 23]. Given the huge socio-economic, cultural and demographic differences between developed countries and LMICs like Nigeria, it cannot be assumed that interventions that are effective in developed countries would be equally effective in settings such as Nigeria. This study therefore assessed the feasibility of parent-mediated behavioural intervention for challenging behaviour in a clinical population of children with ASD in Lagos, South West Nigeria.

## Methods

### Participants and sampling

The participants comprised children with a diagnosis of autism spectrum disorder and their respective mothers. The inclusion criteria were children below the age of 18 years, with a history of aggressive and self-injurious behaviour and attending the Neurodevelopmental Clinic at Child and Adolescent Mental Health Service Unit of Federal Neuro-Psychiatric Hospital (FNPH), Lagos, Nigeria, and whose mothers gave consent. The neurodevelopmental clinic is a tertiary centre that receives referrals from other parts of the country.

Using sample size calculation described by Wade [24], 16 mothers was identified as adequate to detect a post-intervention difference of one standard deviation in outcome measures based on 5 % level of significance and 80 % power. The sample was increased to 20 account for possible drop outs. We hypothesized such a large post-intervention difference because the huge treatment gap in Africa increases the likelihood that simple interventions can produce huge outcomes [25].

### Measures

The instrument used for data collection comprised a socio-demographic questionnaire, aggression and self-injury questionnaire (ASIQ), knowledge of behavioural management of aggression questionnaire (KBMAQ) and client satisfaction questionnaire (CSQ). The instrument was pre-tested on 10 mothers of children with ASD and challenging behaviour outside the study population, and found to be comprehensible and reliable for the population of children with ASD. Two weeks test retest reliability for the ASIQ and KBMAQ were excellent (r = 0.95, p < 0.001; r = 0.94, p < 0.001 respectively). Cronbach alphas are 0.86, 0.87, and 0.81 for ASIQ, KBMAQ, and CSQ respectively.

The socio-demographic questionnaire obtained information on participants’ socio-demographic details such as age, gender, marital status and level of education.

Aggression and self injurious behaviour questionnaire (ASIQ) was adapted by the first author from Hyman et al. [26] and Rojahn et al. [27]. The questionnaire has two sections. The first section has 12 items that assess aggressive behaviour against a person or property (APP section). The second Section has 10 items that measure self-injurious behaviours (SIB section). Each item on the APP and SIB sections was scored on four scales: a five-point frequency scale (never = 0, monthly = 1, weekly = 2, daily = 3, and two or more times daily = 4), a four-point severity scale (0 = no problem, 1 = slight problem, 2 = moderate problem, and 3 = severe problem), a five-point duration scale (1 = <1 min, 2 = <5 min, 3 = <15 min, 4 = <1 h, and 5 = 1 h or more), and finally a five-point need for physical restraint scale (0 = never, 1 = at least once a month, 2 = at least once a week, 3 = at least once a day, and 4 = at least once an hour while awake). A total score was obtained for each item by summing the scores on all the four domains for that item: frequency, severity, intensity and physical restraint domains. On this instrument a higher score indicate a more difficult or severe challenging behaviour. The items were completed at baseline and post-intervention by a trained interviewer who was blind to the aim of the intervention.

Knowledge of behavioural management of aggression questionnaire (KBMAQ) is a 12-item instrument designed by the first and second authors to assess the mothers’ knowledge of the content of the sessions pre and post-intervention. Face validity of this measure was assessed through peer review. Examples of items on the measure include, “For a child who is unable to explain things, the purpose of a challenging behaviour can be identified by examining what he/she was doing before the behaviour started”, “Understanding how a challenging behaviour ends can help to identify how to prevent it in future”. Each item on the measure was scored on a scale of “true”, “false” and “don’t know”. One mark was given for a “true” response (correct answer) and a zero for either a “false” (incorrect answer) or “don’t know” option. The total possible score on this measure ranged from 0 to 12 with a higher score indicating a higher level of knowledge.

The Client satisfaction questionnaire consists of 8 questions modified from Attkinson and Greenfied [28], to assess the mothers’ satisfaction with the programme post-intervention. Each question is scored on a Likert scale of 1–4 with a total score ranging from 8 to 32. On this instrument, a higher score indicates a higher level of satisfaction. The instrument has been found to be reliable for use in Nigeria with a Cronbach alpha of 0.81 [29].

The study instruments were translated into Yoruba by a Yoruba speaking psychiatrist and a linguist. The back translation was performed independently by another psychiatrist and another linguist. This back translation was then compared with the original translation by an independent panel and confirmed to be satisfactory before use.

### The intervention

The Behavioural intervention manual for aggression in ASD used for this study was adapted by the second author from previous works including Durand and Crimmins [30] and Iwata and Dozier [31]. The intervention was delivered by the first author who is a consultant psychiatrist with training in behavioural interventions in ASD. The other authors provided supervision. The intervention was delivered in a group format as this is likely to be more cost-effective in a low and middle income country (LMIC) such as Nigeria.

The behavioural intervention comprised five workshop-styled sessions that includes interactive group discussion and problem solving. The first session introduced concepts such as ASD, associated impairments and aggression in ASD. The second session explained the basic principles of functional behaviour analysis (FBA) for aggression in ASD and identification of triggers. The third session focussed on the principle of contingency management such as use of reward to encourage more adaptive behaviours and non-physical consequences to reduce aggression. The fourth session was a further extension/reinforcement of the issues covered in the second and third sessions. This helped to embed the concepts and address practical issues arising from each mother’s use of the strategies with their own children. The fifth session was a review of the four previous sessions.

### Study procedure

The study procedure was in three stages. The first stage involved making or re-confirming a diagnosis of Autism Spectrum Disorder. The first author, a Consultant Psychiatrist in Child and Adolescent Mental Health, carried out a psychiatric assessment on every child with a previous diagnosis of ASD and any new patients suspected to have the disorder. The clinical diagnosis of ASD was based on DSM-5 criteria [4].

Secondly, the mothers of children with ASD, who met the inclusion criteria, were interviewed individually in separate rooms. The socio-demographic questionnaire, aggression questionnaire and knowledge questionnaire were administered to the mothers by a trained interviewer who was blind to the study hypotheses. Subjects who were unable to communicate in English language were interviewed in Yoruba language. The instruments were translated from English into Yoruba Language to facilitate easy comprehension by participants who were unable to communicate in English Language.

The third stage involved delivery of the five sessions of intervention. This was done weekly in a group format with ten mothers in each group. In between sessions, mothers were contacted via telephone calls and short message service (SMS) to assist with problem-solving and to remind them of the date of next intervention session. The 20 mothers attended all the intervention sessions and completed all the outcome measures. Post intervention assessments were conducted a week after the final session. The post-intervention measures were administered by the same trained interviewer who was still blind to the study hypotheses.

### Ethical considerations

The study was approved by the Ethical and Research Committee of the Federal Neuro-Psychiatric Hospital, Yaba, Lagos. Informed consent was obtained from all the mothers after an explanation of the aim of the study. Informed consent of fathers was also obtained; either directly from those fathers who accompanied the child to the clinic or indirectly over the phone. Assent was obtained from children with ASD who were judged to be competent.

### Data analysis and management

Data were analysed with Statistical Package for Social Sciences software version 21. Categorical socio-demographic variables and types of aggressive behaviour were presented as frequencies and proportions. Continuous measures such as age, APP, SIB, and KBMAQ were presented as mean and standard deviations. Differences in pre and post-intervention scores on non-normally distributed outcome measures (APP and SIB) were analysed with Wilcoxon signed-rank test and paired *t* test for KBMAQ.

## Results

A total of 20 children with a diagnosis of Autism Spectrum Disorder (ASD) and their respective mothers participated in this study. All the children with ASD had aggressive and self injurious behaviour.

Table 1 shows the socio-demographic characteristics of the children with ASD and their mothers. The children were aged 3–17 years (mean = 10.7 years, SD 4.6 years), while their mothers’ age ranged from 32 to 52 years (with a mean age of 42.8 years, SD, 6.4). There was a male preponderance (65.0 %) among the children in the sample. 55 % of the children were in special schools that were non-specific for autism while almost a third (30.0 %) was out of school (Table 1). 85 % of the mothers were currently married, and a similar proportion had a minimum of 12 years formal education (Table 1).

**Table 1:** Socio-demographic characteristics of study participants (children with ASD and mothers) N = 20

Variable	Frequency (n)	Percentage (%)
Child gender		
Male	13	65.0
Female	7	35.0
Child’s education		
Special school	11	55.0
Out of school	6	30.0
Mainstream school	3	15.0
Birth order		
First child	8	40.0
Middle child	5	25.0
Last child	7	35.0
Marital status		
Currently married	17	85.0
Separated/divorced	2	10.0
Widowed	1	5.0
Family setting		
Monogamous	15	75.0
Polygamous	5	25.0
Mother’s education		
6 years of formal education	3	15.0
12 years of formal education	5	25.0
Tertiary education	12	60.0
Religion		
Christianity	15	75.0
Islam	5	25.0

Of the measured 12 items on aggressive behaviour towards a person or property (APP) category of ASIQ, destructiveness had the highest rate of 65.0 %, followed by hitting and pulling with a rate of 55.0 %. Of the 10 items measured on the self injurious behaviour (SIB) category of ASIQ, self-hitting with hand was the most frequent at a rate of 50.0 %, followed by self-biting (45.0 %). Tables 2 and 3 show Wilcoxon signed-rank test for the differences in the pre and post intervention scores on the APP and SIB measures. There was a statistically significant reduction in the post intervention scores on all the four domains of aggression towards APP compared to pre-intervention scores (Table 2).

**Table 2:** Differences between pre and post intervention outcome measures for aggressive behaviour towards a person or property in children with ASD N = 20

Variable	Pre-intervention	Post-intervention	Wilcoxon rank	p
	Median (interquartile range)	Median (interquartile range)		
APP frequency	11.0 (11.0)	9.0 (13.0)	−2.560	0.010*
APP severity	7.0 (8.0)	6.5 (8.0)	−2.507	0.012*
APP duration	7.5 (9.0)	5.0 (10.0)	−2.825	0.005*
APP need for physical restraint	8.5 (7.0)	5.5 (8.0)	−3.346	0.001*
APP total score	32.5 (30.0)	26.5 (36.0)	−3.519	<0.001*

* Significant at p < 0.05

**Table 3:** Differences between pre and post intervention outcome measures for self-injurious behaviour (SIB) in children with ASD N = 20

Variable	Pre-intervention	Post-intervention	Wilcoxon rank	p
	Median (interquartile range)	Median (interquartile range)		
SIB frequency	11.0 (12.0)	10.0 (11.0)	−2.967	0.003*
SIB severity	7.0 (7.0)	6.5 (6.0)	−2.414	0.016*
SIB duration	5.5 (6.0)	5.0 (6.0)	−2.232	0.026*
SIB need for restraint	7.5 (10.0)	7.0 (8.0)	−2.549	0.011*
SIB total score	33.0 (30.0)	31.0 (30.0)	−2.714	0.007*

* Significant at p < 0.05

The self-injurious behaviour category showed a statistically significant decrease in the post-intervention SIB mean scores compared with pre-intervention scores in all the four domains (Table 3).

The mother’s post-intervention knowledge of the subjects covered in the intervention was statistically significantly higher than their pre-intervention knowledge (Table 4).

**Table 4:** Differences in the pre and post intervention mean scores on knowledge of mothers on behavioural management of aggression in ASD N = 20

Variable	Pre-intervention	Post-intervention	Mean difference (SD)	t	p
	Mean (SD)	Mean (SD)			
Knowledge	7.90 (2.57)	11.80 (0.41)	1.40 (1.19)	5.272	<0.001*

^*^Significant at p < 0.05

The client satisfaction questionnaire showed that the intervention was very well received by the mothers. Two-third (40 %) of mothers rated the intervention programme as good while 60 % rated it as excellent. The majority (85 %) of mothers endorsed that the programme helped them cope a lot better with their child’s problem behaviour. 80 % of the mothers were very satisfied, and all (100 %) would recommend it to a friend whose child has a similar problem.

## Discussion

Studies from developed countries have shown that behavioural problems in ASD can be effectively managed with parent-delivered behavioural interventions [12, 32, 33]. This feasibility study suggests that parents of children with ASD and challenging behaviour in resource-poor settings like Nigeria can understand and use behavioural intervention to reduce disruptive behaviour in their children. To our knowledge, this is the first study in sub-Saharan Africa to show that a behavioural intervention for challenging behaviour in ASD based on FBA is feasible in this part of the world.

This study adds to the existing evidence of the potential benefit of parent-mediated behavioural intervention for problem behaviour in ASD. For example, in a Canadian study conducted in a community day-care centre over 12 weeks, Jocelyn et al. [12] taught 35 parents the use of functional analysis to understand challenging behaviour in children with ASD and developed treatment strategies for managing such behaviours. They found significant improvements in post test behavioural measures. In another study using reinforcement, antecedent—based techniques and environmental manipulations, Butler and Luselli [34] demonstrated a reduction in aggression to near zero level among children with autism aged 1–13 years. Similarly, Frea et al. [35] reported an immediate and rapid reduction in aggression in children with autism and intellectual disability through the use of picture exchange communication system (PECS) while Mueller et al. [36] observed a decrease in aggressive behaviour in children with ASD by active antecedent manipulation of reinforcers. Braithwaite and Richdale [31] and Athens and Vollmer [33] also used reinforcement-based strategies in a behavioural intervention for aggressive behaviour and documented a significant reduction in the rate of aggressive behaviour post intervention.

The finding of the present study is also in line with a large scale randomized clinical trial, conducted by Bearss et al. [37], among 180 children aged 3–7 years with ASD and behavioural problems in the United States. The investigators randomized children and their mothers into two groups to receive either parent training or education aimed at examining the effect of either intervention on disruptive behaviour in their children with ASD. Bearss et al. [37] reported a reduction in disruptive behaviour post behavioural intervention, especially in the parent training group.

These findings support the effectiveness of behavioural programmes that include identification of the functions of challenging behaviour, and developing a behavioural plan that specifies strategies to alter the antecedents and reduce the contingencies that increase the behaviour while enhancing those that terminate or reduce the challenging behaviour. The robustness of this evidence underlines its recommendation in guidelines for management of children with ASD [17].

However, while the principles of behavioural intervention based on FBA are now well established, putting them into practice especially with parents with a priori limited knowledge of ASD or behavioural psychology or even basic literacy can be a challenge. Nonetheless, this study shows that such an intervention is feasible even in resource-poor settings like Nigeria, in so far as the programme is explained at a level accessible to parents. It suggests that parents in these settings can understand it and put the techniques into practice, and report significant reductions in their childrens’ challenging behaviour. The study also suggests that the intervention was highly acceptable to the parents with the vast majority being very satisfied and all participants willing to recommend it to a friend whose child has similar difficulties. The fact that the improvements were reported with a relatively short intervention of five sessions is particularly encouraging because brief interventions are more likely to be feasible in resource-limited settings like Nigeria. The use of a group format, which could be cheaper than individualised intervention in a poor resource setting, adds further to the feasibility.

Another important observation from the study is that about a third of the children were out of school and all those in special schools were in settings not specialized for the specific need of children with ASD. This is consistent with previous studies in the country [3, 38]. Omigbodun [38] found that 27.6 % of the children with ASD in Ibadan, Nigeria were out of school due to lack of suitable schools to meet their educational needs. Similarly, Bello-Mojeed et al. [3] reported that 41 % of Nigerian children with ASD had no access to formal education while 69 % of those in contact with educational setting were out of school. These findings highlight the serious barriers encountered in accessing appropriate educational placement for Nigerian children with ASD. One possible explanation is that lack of skills in managing ASD-related challenging behaviour may be preventing mainstream schools from admitting children with ASD whose educational needs might otherwise be met within inclusive educational settings. This suggests that extending behavioural interventions for managing aggression to Nigerian teachers could improve access to education for the large number of children with ASD who are currently without any educational placement.

While the findings of this study are promising, they need to be interpreted with some limitations in mind. The main limitation is the lack of a control group. This means that the improvements noted could be attributable to other factors unrelated to the intervention such as regression to the mean, practice effect, attention, and or the enthusiasm of the workshop leader. Similarly, lack of independent rating of outcomes means that the mothers may have subconsciously reported positive outcomes to justify the investment in time and energy they made to attend the programme. However, the significant improvement in the mothers’ knowledge of the themes covered in the intervention suggests that some of the benefits could be related to the intervention. The relatively small sample size which was also selected from a tertiary referral centre makes it difficult to generalize the findings to the general population of children with ASD and aggression in Nigeria or sub-Saharan Africa. The duration of the post-intervention outcome assessment was short and this makes it difficult to evaluate the long term effect of the intervention.

## Conclusions

This study suggests that challenging behaviour in children with ASD in a resource-poor setting like Nigeria could be significantly reduced with a brief (5 sessions) behavioural intervention based on FBA delivered by parents with the support of a professional. This suggests that FBA-based behavioural intervention is feasible and shows some promise as an effective treatment option for reducing challenging behaviour in children with ASD in Nigeria and other LMICs. Future studies in LMICs should explore the efficacy of this intervention with randomised controlled trials using independently rated outcome measures with some masking. We recommended that future studies use standardised outcome measures with clinical cut-offs so that the clinical significance of any changes can be identified.

## Abbreviations

ABA: applied behaviour analysis
ABC: antecedent behaviour consequence
APP: aggression towards a person or property
ASIQ: aggression and self injurious questionnaire
APA: American Psychiatric Association
APP: aggression against a person or property
ASD: Autism Spectrum Disorders
ASIQ: aggression and self injurious behaviour questionnaire
CSQ: client satisfaction questionnaire
DSM V: diagnostic and statistical manual of mental disorder version v
FBA: functional behaviour analysis/assessment
FNPH: federal neuro-psychiatric hospital
KBMAQ: knowledge on behavioural management of aggression questionnaire
LMIC: low and middle income countries
NICE: National Institute for Health and Clinical Excellence
PECS: picture exchange communication system
SIB: self injurious behaviour
SMS: short message service
